# Influence of Indole-3-Acetic Acid and Gibberellic Acid on Phenylpropanoid Accumulation in Common Buckwheat (*Fagopyrum esculentum* Moench) Sprouts

**DOI:** 10.3390/molecules22030374

**Published:** 2017-02-28

**Authors:** Chang Ha Park, Hyeon Ji Yeo, Yun Ji Park, Abubaker M. A. Morgan, Mariadhas Valan Arasu, Naif Abdullah Al-Dhabi, Sang Un Park

**Affiliations:** 1 Department of Crop Science, Chungnam National University, 99 Daehak-Ro, Yuseong-gu, Daejeon 34134, Korea; parkch804@gmail.com (C.H.P.); guswl7627@naver.com (H.J.Y.); yunji0825@hanmail.net (Y.J.P.); abubaker_morgan@yahoo.com (A.M.A.M.); 2 Department of Botany and Microbiology, Addiriyah Chair for Environmental Studies, College of Science, King Saud University, P.O. Box 2455, Riyadh 11451, Saudi Arabia; mvalanarasu@gmail.com (M.V.A.); naldhabi@ksu.edu.sa (N.A.A.-D.)

**Keywords:** indole-3-acetic acid, gibberellic acid, phenylpropanoid, common buckwheat, *Fagopyrum esculentum* Moench

## Abstract

We investigated the effects of natural plant hormones, indole-3-acetic (IAA) acid and gibberellic acid (GA), on the growth parameters and production of flavonoids and other phenolic compounds in common buckwheat sprouts. A total of 17 phenolic compounds were identified using liquid chromatography-mass spectrometry (LC-MS) analysis. Among these, seven compounds (4-hydroxybenzoic acid, catechin, chlorogenic acid, caffeic acid, epicatechin, rutin, and quercetin) were quantified by high-performance liquid chromatography (HPLC) after treating the common buckwheat sprouts with different concentrations of the hormones IAA and GA. At a concentration of 0.5 mg/L, both IAA and GA exhibited the highest levels of growth parameters (shoot length, root length, and fresh weight). The HPLC analysis showed that the treatment of sprouts with IAA at concentrations ranging from 0.1 to 1.0 mg/L produced higher or comparable levels of the total phenolic compounds than the control sprout and enhanced the production of rutin. Similarly, the supplementation with 0.1 and 0.5 mg/L GA increased the content of rutin in buckwheat sprouts. Our results suggested that the treatment with optimal concentrations of IAA and GA enhanced the growth parameters and accumulation of flavonoids and other phenolic compounds in buckwheat sprouts.

## 1. Introduction

*Fagopyrum esculentum* Moench, also called common buckwheat, is mainly cultivated and consumed in China, Japan, and South Korea [1]. Common buckwheat is considered an important pseudocereal owing to its medicinal and agricultural values. It has various allelopathic [2] and medicinal (i.e., anti-hypertensive [3], anti-allergic [4], antibacterial [5], antioxidant [6], and cytotoxic [7]) properties. These properties are attributed to various nutrients, such as vitamins, amino acids, fatty acids, dietary fibers, minerals, phenolic compounds, and flavonoids, present in common buckwheat [2,3].

Flavonoids are a large group of benzo-γ-pyrone derivatives synthesized in the phenylpropanoid pathway, and are distributed ubiquitously in plant species [8]. They are mainly involved in plant development, growth, pigment generation, and protection against pathogen attack and high light and ultraviolet radiation [9,10]. Previous studies have reported that common buckwheat contains a variety of flavonoids (vitexin, isovitexin, orientin, isoorientin, and rutin) [11], a flavonol (quercetin), flavan-3-ols ((+)-catechin and (−)-epicatechin), and other phenolic compounds (chlorogenic acid, caffeic acid, ferulic acid, and gallic acid) [2]. In particular, rutin (quercetin-3-*O*-rutinoside), a flavonol glycoside, the primary component of the flavonoids present in common buckwheat [2,11,12,13,14] and present in other buckwheat species (*F. homotropicum*, *F. esculentum*, and *F. tataricum*), exhibits various health benefits, including antioxidant [15], anti-diabetic [16], anti-inflammatory [17], anti-neuroprotective [18], and cytotoxic effects [19], as well as inhibition of cardiac hypertrophy [20]. Additionally, buckwheat is considered a good source of dietary rutin since no previous reports have documented the detection of rutin in other cereals and pseudocereals.

Plant hormones, also called phytohormones, are defined as organic molecules affecting plant physiological processes, including differentiation, development, and growth, at low concentrations [21]. In plant tissue and cell culture, phytohormones were classified into five main classes [auxins, abscisic acid (ABA), cytokinins, ethylene, and gibberellins (GAs)]. Although an interaction between auxins and cytokinins is mainly required to regulate the normal physiological processes in plant tissue and organ cultures, ABA, ethylene, and GAs play regulatory roles [22]. More recently, brassinosteroids, jasmonates, polyamines, and salicylate have been recognized as new classes of plant hormones due to their regulatory roles in plant development [23].

Various approaches, including culture systems, elicitation, plant hormones, nutrient conditions, removal of toxic products, and precursor feeding, have been used to promote the production of secondary metabolites [24,25,26,27,28]. In particular, plant hormones have enhanced the production of specialized metabolites; for example, 6-benzyladenine enhanced rutin concentrations in buckwheat plantlets [29] and ABA upregulated the production of phenolic compounds and glucosinolates in Chinese cabbage [30] and anthocyanins and flavonols in muscadine grapes (*Vitis rotundifolia* Michx.) [31]. Ethylene increased the accumulation of alkaloids in cell cultures of *Catharanthus roseus* (L.) G. Don [32]. The optimized indolebutyric acid concentration led to the enhanced production of periplocin in the adventitious roots of *Periploca sepium* Bunge [33]. Synthetic auxins (2,4-dichlorophenoxyacetic acid and naphthaleneacetic acid) and natural auxins (indole-3-butyric acid and indole-3-acetic acid (IAA)) increased the production of anthocyanins in hairy root cultures of tartary buckwheat cultivar Hokkai T10 [34]. Similarly, the addition of salicylate markedly promoted mulberroside A production in cell suspension and root cultures of *Morus alba* [35] and enhanced the accumulation of alkaloids in hairy roots of *Brugmansia candida* [36]. Furthermore, sorghum (*Sorghum bicolor*) roots, after treatment of jasmonates, exhibited increased accumulation of sorgoleone [37].

Recently, Lee et al. (2014) [38] and Seo et al. (2015) [39] reported the effect of light-emitting diode lights on phenolic compound production in tartary and common buckwheat sprouts. Furthermore, Nam et al. (2015) [40] identified the six flavonoids including vitexin, isovitexin, orientin, isoorientin, rutin, and quercetin-3-*O*-robinobioside in common buckwheat sprouts. In particular, the newly identified quercetin-3-*O*-robinobioside was present only in common buckwheat sprouts. To our knowledge, no previous reports have documented the influence of IAA and GA on the accumulation of flavonoids in common buckwheat sprouts. Thus, the current study aimed to optimize the concentration of IAA and GAs for the growth and production of flavonoids and other phenolic compounds in common buckwheat sprouts using liquid chromatography-mass spectrometry (LC-MS) and high-performance liquid chromatography (HPLC) analyses.

## 2. Results and Discussion

### 2.1. Growth Patterns of Buckwheat Sprouts after Treatment with IAA and GA

**Figure 1:**
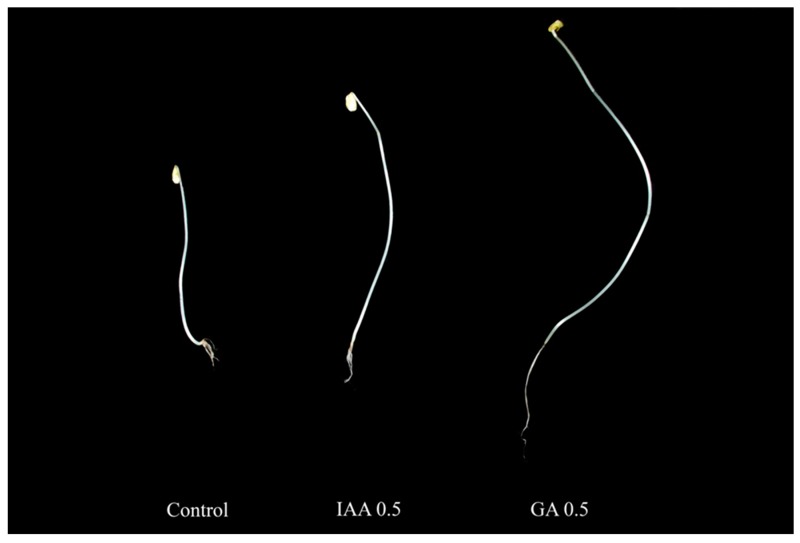
Effect of indole-3-acetic (IAA) and gibberellic acid (GA) on sprout growth.

The growth patterns of buckwheat sprouts supplemented with 0.1, 0.3, 0.5, 3.0, 5.0 mg/L of IAA and GA were assessed by measuring their shoot height (cm), root length (cm), and fresh weight (g). Table 1 shows that the growth patterns of sprouts after treatment with both hormones were almost similar. The supply of exogenous IAA and GA gradually increased the shoot height, root length, and fresh weight of buckwheat sprouts at the concentrations of 0.1, 0.5, and 1.0 mg/L, but the growth patterns were significantly decreased at the concentrations of 3.0 and 5.0 mg/L. We also used an experimental control in which the common buckwheat sprouts were grown without any hormones for 10 days. The treatment of GA at 0.5 mg/L exhibited the highest values of all growth patterns. The values of shoot height, root length, and fresh weight were 1.91, 1.98, and 2.22 times higher than those of the control, respectively. Furthermore, these values were 8.22, 5.24, and 5.00 times higher than the lowest value acquired after treatment with 5.0 mg/L GA. Treatment with 0.5 mg/L IAA resulted in the highest values of all growth patterns, which were 1.79, 3.37, and 2.33 times higher than those of the control, and 5.96, 4.69, and 3.50 times higher than the lowest values after treatment with 5.0 mg/L IAA. Among the different concentrations of IAA and GA, 0.5 mg/L was the most effective concentration for sprout growth (Figure 1).

### 2.2. LC-MS and HPLC Analysis of Common Buckwheat Sprouts after Treatment with IAA and GA

A total of 17 phenolic compounds were identified in common buckwheat sprouts by LC-MS analysis as shown in Table 2. Compounds **1** to **14** exhibited the major ion peaks at *m*/*z* 169.5 [M − H]^−^, 137.3 [M − H]^−^, 289.2 [M − H]^−^, 353.0 [M − H]^−^, 167.9 [M − H]^−^, 179.7 [M − H]^−^, 289.5 [M − H]^−^, 163.8 [M − H]^−^, 193.5 [M − H]^−^, 609.8 [M − H]^−^, 301.9 [M − H]^−^, 285.3 [M − H]^−^, 609.9 [M − H]^−^, and 431.6 [M − H]^−^. Compound **15** showed a major ion peak at *m*/*z* 175.6 [M − Na]^−^. Subsequently, compounds **16** and **17** showed major ion peaks at *m*/*z* 341.8 [M − H]^−^ and 577.7 [M − H]^−^, respectively. 

In the HPLC analysis, seven phenolic compounds were identified and quantified in the buckwheat sprouts supplemented with exogenous hormones (Table 3). The treatment of buckwheat sprouts with IAA at the specific concentrations of 0.1, 0.5, and 1.0 mg/L resulted in an increase in the accumulation of phenolic compounds; however, at 3.0 and 5.0 mg/L, IAA treatment led to a significant decrease in the accumulation of phenolic compounds. Subsequently, the highest level of total phenolic compounds was recorded after supplementing the sprouts with 0.1 mg/L IAA (1580.49 ± 11.19 μg/g Dry Weight (DW)), which was 1.83 times higher than the lowest one (861.74 ± 47.37 μg/g DW). Particularly, the levels of catechin, caffeic acid, and rutin were significantly increased after treatment with 0.1–1.0 mg/L IAA. The highest levels of catechin and caffeic acid were obtained after treatment with 0.5 mg/L IAA, which were 1.24 and 1.63 times higher than the equivalents of the control, respectively. Furthermore, the accumulation of rutin in the sprouts treated with 1.0 mg/L IAA was 1.39 times higher than that in the control. However, the IAA treatment led to a gradual concentration-dependent decrease in the accumulation of 4-hydroxybenzoic acid, chlorogenic acid, epicatechin, benzoic acid, and quercetin. Similarly, the accumulation of catechin, caffeic acid, and rutin was enhanced by supplementing the sprouts with 0.1 to 1.0 mg/L GA. After treating the sprouts with 0.1 mg/L GA, the highest values of catechin, caffeic acid, and rutin were 115.93 ± 5.69, 27.77 ± 1.07, and 866.13 ± 37.41 μg/g DW, respectively, which were 1.26, 1.72, and 1.23 times higher than those of the control. However, the treatment with GA showed a gradual concentration-dependent decrease in the values of the total phenolic compounds.

Both natural and synthetic auxins are important phytohormones which regulate plant development. In particular, IAA are a major plant hormone and its key roles in cell division, elongation, and differentiation are apparently evident from observing the embryonic and post-embryonic development and tropisms (i.e., movement towards gravity and light) [41]. GAs, initially synthesized from the trans-geranylgeranyl diphosphate, are bioactive compounds involved in diverse processes throughout plant growth and development (i.e., promotion of leaf and stem growth, induction of seed germination, modulation of flowering time, and modulation of the development of flowers, fruits, and seeds) [42,43].

Previous studies have reported that the exogenous supply of IAA and GA is a useful approach to enhance the production of various secondary metabolites. The results from these studies were consistent with our findings. Exogenously supplied IAA at 0.1 mg/L enhanced the production of shikonin in cell cultures of *Onosma paniculatum* L. [44]. An exogenous supply of 0.5 and 1.0 mg/L IAA led to an increase in the production of total phenolics and flavonoids in the adventitious roots of *Hypericum perforatum* L. [45]. Similarly, a relatively low concentration of IAA promoted the production of phenolic compounds in the hairy roots of *Panax ginseng* C. A. Meyer [46]. Additionally, the accumulation of aryltetralin-type lignans (e.g., podophyllotoxin and 6-methoxypodophyllotoxin) was increased by the addition of IAA in the hairy root clone of *Linum album* LYR2i [47]. Bais et al. (2001) reported that the exogenous treatment of GA_3_ induced an increase in root growth and the production of coumarins (e.g., esculin and esculetin) in the hairy roots of *Cichorium intybus* L. ‘Lucknow Local’ [48]. Furthermore, GA_3_ enhanced the production of phenolics (e.g., anthocyanin, cichoric acid, caftaric acid, chlorogenic acid, and caffeic acid) in the hairy roots of *Echinacea purpurea* (L.) Moench and sesquiterpenes (e.g., artemisinin) in the hairy roots of *Artemisia annua* L. [49,50].

This study reported that the effect of IAA and GA on the growth and accumulation of phenolic compounds, including rutin and catechin, in common buckwheat sprouts, indicating that the treatment of the natural plant hormones at certain concentrations enhanced the growth and the accumulation of the natural products in the buckwheat sprouts.

## 3. Materials and Methods

### 3.1. Plant Materials

Seeds of common buckwheat were purchased from BONGPYEONG MAEMIL TUEKSANDANGI (Pyeongchang gun, Gangwon-do, Korea). For the treatment, stock solutions of IAA and GA at a concentration of 1 mg/mL was prepared in absolute ethanol, followed by dilution with deionized water to 0.1, 0.5, 1.0, 3.0, and 5.0 mg/L, respectively. 200 seeds (about 4 g) were soaked in tap water for 16 h at 28 °C. Subsequently, the seeds were loaded onto each pot containing vermiculites at 25 °C for germination and immediately treated with natural plant hormones (IAA and GA) at different concentrations (0.1, 0.5, 1.0, 3.0, and 5.0 mg/L) under dark condition in a plant growth chamber. After 2 days, the sprouts started to appear from the surface of the soil and were allowed to grow for 10 days. The sprout samples were frozen using −196 °C liquid nitrogen, and subsequently stored at −80 °C until use. After the lyophilization and grinding process, each sample was weighed to 100 mg and put in a 15-mL conical tube for further analysis.

### 3.2. Chemical and Standards

Both gibberellic acid and indole-3-acetic acid were purchased from MB Cell, Seoul, Korea. External standards were purchased from different sources: hydroxybenzoic acid from Extrasynthese (Genay, France) and benzoic acid, catechin, caffeic acid, chlorogenic acid, epicatechin, quercetin, and rutin from Sigma-Aldrich Co., Ltd. (St. Louis, MO, USA).

### 3.3. Extraction and Analysis of Flavonoids

The extraction and analysis of flavonoids in common buckwheat sprouts were carried out using the slightly modified protocol of Li et al. (2014) [51]. For flavonoid extraction, 5 mL of aqueous methanol (80 % *v/v*) containing 10% acetic acid (0.1% *v/v*) was added into the conical tube containing the sample powder (100 mg). Subsequently, it was strongly sonicated at 28 °C for 10 min and extracted in a water bath set at 37 °C for 1 h. Next, the tube was centrifuged at 3000 rpm for 10 min, and the supernatant was transferred to a fresh tube. The entire procedure was performed twice. The collected supernatant was dried using nitrogen gas, before being resuspending in 5 mL of methanol. Additionally, the extracts were diluted by half with the addition of methanol, followed by their filtration and storage in a brown vial. Each flavonoid was separated using an HPLC system (NS-4000, Futecs, Daejeon, Korea) equipped with a C_18_ column (250 mm × 4.6 mm, 5 μm; RStech, Daejeon, Korea), UV-Vis detector, and an auto-sampler. The analysis was performed under the controlled conditions (detection wavelength, 280 nm; flow rate, 1.0 mL/min; injection volume, 20 μL; and column temperature, 30 °C). The HPLC mobile phase was composed of a mixture of (A) acetic acid/water (0.15:99.85, *v/v*), and (B) methanol. The gradient program were set as follows: The gradient conditions were established as follows: 0–1.0 min, 95% A; 1.1–4.0 min, 95%–85% A; 4.1–9.0 min, 85% A; 9.1–14.0 min, 85%–80% A; 14.1–24.0 min, 80% A; 24.1–54.0 min, 80%–70% A; 54.1–55.0 min, 70%–55% A; 55.1–65.0 min, 55% A; 65.1–75.0 min, 55%–44% A; 75.1–77.0 min, 44%–40% A; 77.1–79.0 min, 40% A; 79.1–80.0 min, 40%–20% A; 80.1–90.0 min, 20% A; 90.1–91.0 min, 20%–95% A and 91.1–98.0 min, 95% A. The flavonoid contents were determined according to the retention time and spiking test, followed by a calculation using the equivalents of the external standard chemicals. The SAS software (version 9.4, 2013; SAS Institute, Inc., Cary, NC, USA) was used for analysis of variance (ANOVA) evaluation and Duncan’s Multiple Range Test (DMRT) at *p* < 0.05. The HPLC results were expressed as microgram per milligram dry weight (μg/g DW) with the means ± standard deviation of triplicate experiments.

### 3.4. HPLC-MS Analysis of Flavonoids

The control sprouts, sprouts treated with 0.1 mg/L GA, and sprouts treated with 1.0 mg/L IAA were used for LC-MS analyses, which were performed on a system consisting of an Agilent 1200 series HPLC (Agilent Technologies, Palo Alto, CA, USA) fitted with a 4000 Qtrap LC/MS/MS system (Applied Biosystems, Foster City, CA, USA) in the negative ion mode ([M − H]^−^, [M − Na]^−^). For the LC-MS conditions, the scan range, scan time, curtain gas, heating gas temperature, nebulizing gas, heating gas, ion spray voltage, declustering potential, and entrance potential were 100–1300 *m*/*z*, 4.80 s, 20.00 psi (N_2_), 550 °C, 50.00 psi, 50.00 psi, 5500 V, 100 V, and 10 V, respectively. The mobile phase and gradient program were set using the same procedure described in Extraction and analysis of flavonoids.

## 4. Conclusions

To the best of our knowledge, this is the first study to identify and quantify phenolic compounds, including rutin, in common buckwheat sprouts supplemented with the natural plant hormones, GA and IAA, at different concentrations. A total of 17 phenolic compounds were identified through LC-MS and seven of those compounds were quantified with HPLC after treatment with IAA and GA at different concentrations. Apparently, IAA and GA at specific concentrations enhanced the growth and the accumulation of total phenolic compounds as well as specific flavonoids, including rutin and catechin, in common buckwheat sprouts. Therefore, it is expected that these findings might help develop efficient strategies to produce common buckwheat sprouts as a good source of dietary rutin for human consumption.

## Figures and Tables

**Table 1:** Morphological differentiation in common buckwheat sprouts treated with exogenous hormones at different concentrations. Data were recorded after 10 days of growth.

Hormones	Concentration (mg/L)	Shoot Length (cm)	Root Length (cm)	Fresh Weight (g)
Control	0	6.77 ± 0.29c ^1^	1.67 ± 0.09d	0.09 ± 0.01de
IAA	0.10	11.63 ± 0.50a	2.50 ± 0.17c	0.17 ± 0.01ab
	0.50	12.10 ± 0.71a	5.63 ± 0.21a	0.21 ± 0.04a
	1.00	10.07 ± 0.35b	3.67 ± 0.14b	0.14 ± 0.04bc
	3.00	5.83 ± 0.10d	1.90 ± 0.11cd	0.11 ± 0.01cd
	5.00	2.03 ± 0.26e	1.20 ± 0.06d	0.06 ± 0.01e
Control	0	6.77 ± 0.29c	1.67 ± 0.09bc	0.09 ± 0.01b
GA	0.10	8.30 ± 0.40b	2.93 ± 0.13a	0.13 ± 0.02b
	0.50	12.90 ± 0.36a	3.30 ± 0.20a	0.20 ± 0.05a
	1.00	8.07 ± 0.64b	2.23 ± 0.13b	0.13 ± 0.00b
	3.00	5.27 ± 0.21d	1.07 ± 0.09cd	0.09 ± 0.01b
	5.00	1.57 ± 0.15e	0.63 ± 0.04d	0.04 ± 0.01c

^1^ Means with different letters (a, b, c, d, and e) in the same column differ significantly (*p* < 0.05, ANOVA, Duncan Multiple Range Test (DMRT)).

**Table 2:** List of detected phenolic compounds and their retention times (*t*_R_), and molecular ([M − H]^−^, [M − Na]^−^) and fragment ions in negative mode in *Fagopyrum esculentum* Moench.

No	Name	Molecular Formula	Molecular Weight	*t*_R_ (min)	[M − H]^−^	[M − Na]^−^
**1**	Gallic acid	C_7_H_6_O_5_	170.12	41.80	169.5	
**2**	4-hydroxybenzoic acid	C_7_H_6_O_3_	138.12	20.10	137.3	
**3**	Catechin	C_15_H_14_O_6_	290.27	20.30	289.2	
**4**	Chlorogenic acid	C_16_H_18_O_9_	354.31	32.60	353.0	
**5**	4-hydroxy-3-methoxybenzoic acid	C_8_H_8_O_4_	168.15	49.60	167.9	
**6**	Caffeic acid	C_9_H_8_O_4_	180.16	36.50	179.7	
**7**	*Epi*-catechin	C_15_H_14_O_5_	290.27	42.20	289.5	
**8**	*P*-coumaric acid	C_9_H_8_O_3_	164.16	84.90	163.8	
**9**	Ferulic acid	C_10_H_10_O_4_	194.18	28.50	193.5	
**10**	Rutin	C_27_H_30_O_16_	610.52	66.23	609.8	
**11**	Quercetin	C_15_H_10_O_7_	302.24	79.71	301.9	
**12**	Kaempferol	C_15_H_10_O_6_	286.23	81.55	285.3	
**13**	Quercetin-3-*O*-neohesperidoside	C_27_H_30_O_16_	610.52	67.11	609.9	
**14**	Apigenin glucoside	C_21_H_20_O_10_	432.38	63.68	431.6	
**15**	Syringic acid	C_9_H_10_O_5_	198.17	59.52		175.6
**16**	Caffeic acid hexose	C_15_H_17_O_9_	341.08	63.50	341.8	
**17**	Procyanidin B2	C_30_H_25_O_12_	578.13	59.70	577.7	

**Table 3:** Effect of IAA and GA on the accumulation of phenolic compounds in common buckwheat sprouts.

Natural Plant Hormones	Concentrations (mg/L)	Phenolic Compounds (μg/g)							
		4-hydroxybenzoic acid	Catechin	Chlorogenic Acid	Caffeic Acid	Epicatechin	Rutin	Quercetin	Total
Control	0	7.02 ± 0.72a ^1^	91.77 ± 7.45b	123.28 ± 9.98a	16.19 ± 3.47b	455.38 ± 50.05a	704.22 ± 21.61c	52.11 ± 4.74a	1449.97 ± 11.19b
IAA	0.1	1.79 ± 0.11d	90.90 ± 11.46b	112.86 ± 15.44ab	18.06 ± 1.95b	356.76 ± 0.27b	977.1 ± 31.68a	23.02 ± 1.23c	1580.49 ± 14.96a
	0.5	0.44 ± 0.27e	113.35 ± 5.26a	100.13 ± 3.78b	26.34 ± 0.21a	327.90 ± 28.95bc	852.46 ± 11.88b	20.23 ± 3.67c	1440.85 ± 10.93b
	1.0	3.98 ± 0.02c	113.01 ± 9.51a	101.79 ± 3.93b	24.03 ± 4.87a	307.31 ± 7.52bcd	982.15 ± 62.65a	45.85 ± 1.70b	1578.12 ± 10.93a
	3.0	1.31 ± 0.22d	84.88 ± 13.16b	97.59± 9.78b	14.48 ± 3.53bc	290.73 ± 34.52cd	495.58 ± 12.06d	18.12 ± 3.02c	1002.69 ± 55.49c
	5.0	5.78 ± 0.74b	89.08 ± 3.75b	74.33 ± 4.63c	9.04 ± 3.84c	261.52 ± 35.80d	414.54 ± 9.40e	7.46 ± 2.10d	861.74 ± 47.43d
Control	0	7.02 ± 0.72a	91.77 ± 7.45c	123.28 ± 9.98a	16.19 ± 3.47b	455.38 ± 50.05a	704.22 ± 21.61c	52.11 ± 4.74b	1449.97 ± 11.19a
GA	0.1	1.28 ± 0.38b	115.93 ± 5.69a	111.85 ± 6.75a	27.77 ± 1.07a	248.27 ± 34.82b	866.13 ± 37.41a	62.27 ± 10.49a	1433.51 ±19.21a
	0.5	0.00 ± 0.00c	103.96 ± 4.71b	114.07 ± 8.30a	27.80 ± 3.02a	251.46 ± 0.75b	756.98 ± 40.04b	70.70 ± 5.12a	1324.97 ± 10.93b
	1.0	1.11 ± 0.33b	103.43 ± 1.94b	97.39 ± 8.88b	24.11 ± 3.53a	266.19 ± 8.06b	644.23 ± 12.04d	31.07 ± 2.19c	1167.53 ± 10.93c
	3.0	0.46 ± 0.80bc	76.96 ± 6.72d	62.60 ± 5.58c	15.06 ± 5.02b	234.31 ± 21.80b	311.49 ± 5.88e	15.26 ± 1.32d	716.14 ± 25.48d
	5.0	0.13 ± 0.23c	96.63 ± 3.27bc	54.13 ± 2.60c	12.43 ± 2.59b	268.42 ± 12.23b	289.61 ± 6.36e	10.59 ± 0.60d	731.94 ± 12.68d

^1^ Means with different letters (a, b, c, d, and e) in the same column differ significantly (*p* < 0.05, ANOVA, DMRT).

## References

[1] Jeon Y.-J., Kang E.-S., Hong K.-W. (2007). A PCR method for rapid detection of buckwheat ingredients in food. J. Korean Soc. Appl. Biol. Chem..

[2] Golisz A., Lata B., Gawronski S.W., Fujii Y. (2007). Specific and total activities of the allelochemicals identified in buckwheat. Weed Biol. Manag..

[3] Kim D.W., Hwang I.K., Lim S.S., Yoo K.Y., Li H., Kim Y.S., Kwon D.Y., Moon W.K., Kim D.W., Won M.H. (2009). Germinated buckwheat extract decreases blood pressure and nitrotyrosine immunoreactivity in aortic endothelial cells in spontaneously hypertensive rats. Phytother. Res..

[4] Kim C.D., Lee W.-K., No K.-O., Park S.-K., Lee M.-H., Lim S.R., Roh S.-S. (2003). Anti-allergic action of buckwheat (*Fagopyrum esculentum* Moench) grain extract. Int. Immunopharmacol..

[5] Amarowicz R., Dykes G.A., Pegg R.B. (2008). Antibacterial activity of tannin constituents from *Phaseolus vulgaris*, *Fagoypyrum esculentum*, *Corylus avellana* and *Juglans nigra*. Fitoterapia.

[6] Ren S.-C., Sun J.-T. (2014). Changes in phenolic content, phenylalanine ammonia-lyase (PAL) activity, and antioxidant capacity of two buckwheat sprouts in relation to germination. J. Funct. Foods.

[7] Danihelová M., Jantová S., Sturdík E. (2013). Cytotoxic and antioxidant activity of buckwheat hull extracts. J. Microbiol. Biotechnol. Food. Sci..

[8] Kumar S., Pandey A.K. (2013). Chemistry and biological activities of flavonoids: An overview. Sci. World J..

[9] Dixon R.A., Paiva N.L. (1995). Stress-induced phenylpropanoid metabolism. Plant Cell.

[10] Winkel-Shirley B. (2001). Flavonoid biosynthesis. A colorful model for genetics, biochemistry, cell biology, and biotechnology. Plant Physiol..

[11] Kim S.-J., Zaidul I., Suzuki T., Mukasa Y., Hashimoto N., Takigawa S., Noda T., Matsuura-Endo C., Yamauchi H. (2008). Comparison of phenolic compositions between common and tartary buckwheat (*Fagopyrum*) sprouts. Food Chem..

[12] Jiang P., Burczynski F., Campbell C., Pierce G., Austria J., Briggs C. (2007). Rutin and flavonoid contents in three buckwheat species *Fagopyrum esculentum*, *F. tataricum*, and *F. homotropicum* and their protective effects against lipid peroxidation. Food Res. Int..

[13] Kalinova J., Triska J., Vrchotova N. (2006). Distribution of vitamin E, squalene, epicatechin, and rutin in common buckwheat plants (*Fagopyrum esculentum* Moench). J. Agric. Food Chem..

[14] Kreft I., Fabjan N., Yasumoto K. (2006). Rutin content in buckwheat (*Fagopyrum esculentum* Moench) food materials and products. Food Chem..

[15] Lee L.-S., Choi E.-J., Kim C.-H., Sung J.-M., Kim Y.-B., Seo D.-H., Choi H.-W., Choi Y.-S., Kum J.-S., Park J.-D. (2016). Contribution of flavonoids to the antioxidant properties of common and tartary buckwheat. J. Cereal Sci..

[16] Watanabe M., Ayugase J. (2010). Effects of buckwheat sprouts on plasma and hepatic parameters in type 2 diabetic db/db mice. J. Food Sci..

[17] Lee C.-C., Shen S.-R., Lai Y.-J., Wu S.-C. (2013). Rutin and quercetin, bioactive compounds from tartary buckwheat, prevent liver inflammatory injury. Food. Funct..

[18] Gulpinar A.R., Orhan I.E., Kan A., Senol F.S., Celik S.A., Kartal M. (2012). Estimation of in vitro neuroprotective properties and quantification of rutin and fatty acids in buckwheat (*Fagopyrum esculentum* Moench) cultivated in Turkey. Food Res. Int..

[19] Kim S.-H., Cui C.-B., Kang I.-J., Kim S.Y., Ham S.-S. (2007). Cytotoxic effect of buckwheat (*Fagopyrum esculentum* Moench) hull against cancer cells. J. Med. Food.

[20] Han S.-Y., Chu J.-X., Li G.-M., Zhu L.-S., Shi R.-F. (2010). Effects of rutin from leaves and flowers of buckwheat (*Fagopyrum esculentum* Moench.) on angiotensin II-induced hypertrophy of cardiac myocytes and proliferation of fibroblasts. Lat. Am. J. Pharm..

[21] Davies P.J., Davies P.J. (2004). The plant hormones: Their nature, occurrence, and functions. Plant Hormones: Biosynthesis, Signal Transduction, Action.

[22] Gaspar T., Kevers C., Penel C., Greppin H., Reid D.M., Thorpe T.A. (1996). Plant hormones and plant growth regulators in plant tissue culture. In Vitro Cell. Dev. Biol. Plant.

[23] Crozier A., Kamiya Y., Bishop G., Yokota T., Buchanan B.B., Gruissem W., Jones R.L. (2000). Biosynthesis of Hormones and Elicitors Molecules. Biochemistry and Molecular Biology of Plants.

[24] Vanisree M., Lee C.-Y., Lo S.-F., Nalawade S.M., Lin C.Y., Tsay H.-S. (2004). Studies on the production of some important secondary metabolites from medicinal plants by plant tissue cultures. Bot. Bull. Acad. Sin..

[25] Cho G., Kim D., Pedersen H., Chin C.K. (1988). Ethephon enhancement of secondary metabolite synthesis in plant cell cultures. Biotechnol. Prog..

[26] Karuppusamy S. (2009). A review on trends in production of secondary metabolites from higher plants by in vitro tissue, organ and cell cultures. J. Med. Plants Res..

[27] Rao S.R., Ravishankar G. (2002). Plant cell cultures: Chemical factories of secondary metabolites. Biotechnol. Adv..

[28] Yeoman M., Yeoman C. (1996). Manipulating secondary metabolism in cultured plant cells. New Phytol..

[29] Hou S., Sun Z., Linghu B., Wang Y., Huang K., Xu D., Han Y. (2015). Regeneration of buckwheat plantlets from hypocotyl and the influence of exogenous hormones on rutin content and rutin biosynthetic gene expression in vitro. Plant Cell Tissue Organ Cult..

[30] Thiruvengadam M., Kim S.-H., Chung I.-M. (2015). Exogenous phytohormones increase the accumulation of health-promoting metabolites, and influence the expression patterns of biosynthesis related genes and biological activity in Chinese cabbage (*Brassica rapa* spp. pekinensis). Sci. Hortic..

[31] Sandhu A.K., Gray D.J., Lu J., Gu L. (2011). Effects of exogenous abscisic acid on antioxidant capacities, anthocyanins, and flavonol contents of muscadine grape (*Vitis rotundifolia*) skins. Food Chem..

[32] Yahia A., Kevers C., Gaspar T., Chénieux J.-C., Rideau M., Crèche J. (1998). Cytokinins and ethylene stimulate indole alkaloid accumulation in cell suspension cultures of *Catharanthus roseus* by two distinct mechanisms. Plant Sci..

[33] Zhang J., Gao W.-Y., Wang J., Li X.-L. (2012). Effects of sucrose concentration and exogenous hormones on growth and periplocin accumulation in adventitious roots of *Periploca sepium* Bunge. Acta Physiol. Plant..

[34] Park C.H., AyeThwe A., Kim S.J., Park J.S., Arasu M., Al-Dhabi N.A., Il Park N., Park S.U. (2016). Effect of Auxins on Anthocyanin Accumulation in Hairy Root Cultures of Tartary Buckwheat Cultivar Hokkai T10. Nat. Prod. Commun..

[35] Komaikul J., Kitisripanya T., Tanaka H., Sritularak B., Putalun W. (2015). Enhanced Mulberroside A Production from Cell Suspension and Root Cultures of *Morus alba* Using Elicitation. Nat. Prod. Commun..

[36] Pitta-Alvarez S.I., Spollansky T.C., Giulietti A.M. (2000). The influence of different biotic and abiotic elicitors on the production and profile of tropane alkaloids in hairy root cultures of *Brugmansia candida*. Enzym. Microb. Technol..

[37] Uddin M.R., Thwe A.A., Kim Y.B., Park W.T., Chae S.C., Park S.U. (2013). Effects of jasmonates on sorgoleone accumulation and expression of genes for sorgoleone biosynthesis in sorghum roots. J. Chem. Ecol..

[38] Lee S.-W., Seo J.M., Lee M.-K., Chun J.-H., Antonisamy P., Arasu M.V., Suzuki T., Al-Dhabi N.A., Kim S.-J. (2014). Influence of different LED lamps on the production of phenolic compounds in common and Tartary buckwheat sprouts. Ind. Crops Prod..

[39] Seo J.-M., Arasu M.V., Kim Y.-B., Park S.U., Kim S.-J. (2015). Phenylalanine and LED lights enhance phenolic compound production in Tartary buckwheat sprouts. Food Chem..

[40] Nam T.-G., Lee S.M., Park J.-H., Kim D.-O., Baek N.-I., Eom S.H. (2015). Flavonoid analysis of buckwheat sprouts. Food Chem..

[41] Teale W.D., Paponov I.A., Palme K. (2006). Auxin in action: Signalling, transport and the control of plant growth and development. Nat. Rev. Mol. Cell Biol..

[42] Sun T.P., Gubler F. (2004). Molecular mechanism of gibberellin signaling in plants. Annu. Rev. Plant Biol..

[43] Hedden P., Phillips A.L. (2000). Gibberellin metabolism: New insights revealed by the genes. Trends Plant Sci..

[44] Yang Y.-H., Huang J., Ding J. (2003). Interaction between exogenous brassinolide, IAA and BAP in secondary metabolism of cultured *Onosma paniculatum* cells. Plant Growth Regul..

[45] Cui X.-H., Chakrabarty D., Lee E.-J., Paek K.-Y. (2010). Production of adventitious roots and secondary metabolites by *Hypericum perforatum* L. in a bioreactor. Bioresour. Technol..

[46] Jeong G.-T., Woo J.-C., Park D.-H. (2007). Effect of plant growth regulators on growth and biosynthesis of phenolic compounds in genetically transformed hairy roots of Panax ginseng CA Meyer. Biotechnol. Bioprocess. Eng..

[47] Farkya S., Bisaria V.S. (2008). Exogenous hormones affecting morphology and biosynthetic potential of hairy root line (LYR2i) of Linum album. J. Biosci. Bioeng..

[48] Bais H., Sudha G., George J., Ravishankar G. (2001). Influence of exogenous hormones on growth and secondary metabolite production in hairy root cultures of *Cichorium intybus* L. cv. Lucknow local. In Vitro Cell. Dev. Biol. Plant.

[49] Abbasi B.H., Stiles A.R., Saxena P.K., Liu C.-Z. (2012). Gibberellic acid increases secondary metabolite production in *Echinacea purpurea* hairy roots. Appl. Biochem. Biotechnol..

[50] Weathers P., Bunk G., McCoy M. (2005). The effect of phytohormones on growth and artemisinin production in *Artemisia annua* hairy roots. In Vitro Cell. Dev. Biol. Plant.

[51] Li X., Kim J.K., Park S.-Y., Zhao S., Kim Y.B., Lee S., Park S.U. (2014). Comparative analysis of flavonoids and polar metabolite profiling of tanno-original and tanno-high rutin buckwheat. J. Agric. Food Chem..

